# Impact of the joint association between sex, age and diabetes on long-term mortality after acute myocardial infarction

**DOI:** 10.1186/s12889-015-1612-x

**Published:** 2015-03-31

**Authors:** Fei Gao, Carolyn Su Ping Lam, Ling Ling Sim, Tian Hai Koh, David Foo, Hean Yee Ong, Khim Leng Tong, Huay Cheem Tan, David Machin, Kok Seng Wong, Mark Yan Yee Chan, Terrance Siang Jin Chua

**Affiliations:** National Heart Centre Singapore, 5 Hospital Drive, Singapore, 169609 Singapore; Centre for Quantitative Medicine, Duke-NUS Graduate Medical School, 8 College Road, Singapore, 169857 Singapore; National University Heart Centre Singapore, National University of Singapore, 1E Kent Ridge Road, Singapore, 119228 Singapore; Cardiac Department, Tan Tock Seng Hospital, 11 Jalan Tan Tock Seng, Singapore, 308433 Singapore; Khoo Teck Puat Hospital, 378 Alexandra Road, Singapore, 159964 Singapore; Changi General Hospital, Simei Street 3, Singapore, 529889 Singapore; Medical Statistics Group, School of Health and Related Sciences, University of Sheffield, Regents Court, 30 Regent Street, Sheffield, S1 4DA UK; Department of Cancer Studies and Molecular Medicine, Clinical Sciences Building, University of Leicester, Leicester Royal Infirmary, Leicester, LE2 7LX UK; Singapore General Hospital, 1 Hospital Drive, Singapore, 169608 Singapore

**Keywords:** Acute myocardial infarction, Diabetes, Women, Young, Mortality

## Abstract

**Background:**

The role of sex, and its joint effect with age and diabetes mellitus, on mortality subsequent to surviving an acute myocardial infarction (AMI) beyond 30 days are unclear. The high prevalence of diabetes mellitus in an ethnically diverse Asian population motivates this study.

**Methods:**

The study population comprised of a nationwide cohort of Asian patients with AMI, hospitalized between 2000 to 2005, who survived the first 30 days post-admission and were followed prospectively until death or 12 years.

**Results:**

Among the 13,389 survivors, there were fewer women (25.5%) who were older than men (median 70 vs. 58 years) and a larger proportion had diabetes mellitus at admission (51.4% vs. 31.4%). During follow-up 4,707 deaths (women 13.2%; men 22.0%) occurred, with women experiencing higher mortality than men with an averaged hazard ratio (HR): 2.08; 95% confidence interval : 1.96-2.20. However the actual adverse outcome, although always greater, reduced over time with an estimated HR: 2.23 (2.04-2.45) at 30 days to HR: 1.75; (1.47-2.09) 12 years later. The difference in mortality also declined with increasing age: HR 1.80 (1.52-2.13) for those aged 22-59, 1.26 (1.11-1.42) for 60-69, 1.06 (0.96-1.17) and 0.96 (0.85-1.09) for those 70-79 and 80-101 years. Significant two-factor interactions were observed between sex, age and diabetes (*P* < 0.001). Diabetic women <60 years of age had greater mortality than diabetic men of the same age (adjusted HR: 1.44; 1.14-1.84; *P* = 0.003), while diabetic women and men *≥*60 years of age had a less pronounced mortality difference (adjusted HR: 1.12; 0.99-1.26).

**Conclusions:**

One in two women hospitalized for AMI in this Asian cohort had diabetes and the sex disparity in post-MI mortality was most pronounced among these who were <60 years of age. This underscores the need for better secondary prevention in this high-risk group.

## Background

Acute myocardial infarction (AMI) is a leading global cause of death in both men and women. Sex, age and diabetes are known determinants of survival following AMI. Data over the past decade have shown that women have a higher 30-day mortality after AMI than men [1,2]. Prior studies suggest that higher early post-MI mortality rates may be limited to younger women, who represent a distinct group characterized by unique risk factors and pathophysiology [3]. The presence of diabetes greatly increases the risk of coronary heart disease (CHD) in both men and women, and diabetic women appear to be at a higher risk of death compared with diabetic men [4-8]. Nevertheless, the extent to which diabetes, female sex and age, interact and impact clinical outcomes over time following AMI remains unclear.

With the marked geo-economic transition in recent decades, the burden of cardiovascular disease has shifted to Asia where it is expected to reach epidemic proportions due to rapidly increasing rates of smoking, obesity and diabetes [9,10]. Yet, epidemiologic data are scarce among Asian populations. In 2007, more than 110 million individuals in Asia were living with diabetes, with a disproportionate burden among the young and middle aged [11]. Because there is considerable heterogeneity in ethnicity, culture, and the stages of socioeconomic development within Asia, which affect clinical presentation, management, and prevention of diabetes [12-15], more studies on diabetic outcomes in economically developed Asian countries will facilitate future comparative studies with the industrialized Western world. We sought to examine the impact of sex and its interaction with age and diabetes on long-term mortality following AMI in a large Asian cohort.

## Methods

### Patient population

Singapore is a densely populated island with a resident population of 5.5 million people and a per capita gross domestic product of 516 USD, among the highest in the world [16]. The public healthcare system provides access for all Singapore citizens and permanent residents through interdependent government-led financing programs, thereby minimizing treatment disparities. The public hospitals manages 80% of acute diseases and 96% of AMI cases in Singapore [17].

Patients were identified from the Singapore Myocardial Infarction Registry (SMIR), a prospective registry of consecutive hospitalizations for AMI at all public hospitals in Singapore. In accordance with Singapore state legislature, collection of clinical data for disease-specific national registries does not require prior informed consent, thereby ensuring an unselected study cohort that is representative of the general population. Such legislature, which is also in effect in many developed Western healthcare systems [18,19], further allows for the linkage of national registry data to national birth and death registries through state-issued identification. The methods of the SMIR have been previously described [20]. Briefly, trained staff at the SMIR perform all data collection, site monitoring and case adjudication. AMI cases are adjudicated using admission and discharge criteria in agreement with the International Classification of Diseases, Ninth Revision codes 410.00 to 414.19, or post-mortem reports.

With the introduction of the universal definition of MI in 2000, we reviewed the troponin and creatine kinase MB (CK-MB) results of all patients in our study population. A total of 87.3% of patients had troponin or CK-MB levels above the 99th percentile of each hospital laboratory's reference population and a diagnosis of AMI was made in these patients if there was accompanying ischemia. The remaining 12.7% did not have sufficient cardiac biomarker data to make a diagnosis based on the universal definition. These patients were diagnosed based on the original World Health Organization-MONItoring trends and determinants of CArdiovascular disease diagnostic criteria at admission [21], Sensitivity analyses in which we removed the 12.29% of cases did not change the directionality of our results.

The study was conducted according to the Helsinki declaration and the National Healthcare Group Domain Specific Review Boards approved the data analysis and collection in all 6 publicly funded hospitals in Singapore.

### Clinical variables

Data regarding patient demographics, medical history, severity of the AMI, and early medical and acute reperfusion therapies were recorded during the index AMI admission at each hospital.

History of diabetes was defined as the presence of a prior diagnosis of diabetes mellitus or current treatment with anti-diabetic drugs. Patients with elevated acute fasting glucose levels without a prior history of diabetes had their fasting glucose levels repeated at 4-8 weeks after discharge. The diagnosis of diabetes was then made in accordance with 1999 WHO criteria (fasting plasma glucose of >7.0 mmol/l or a 2-Hour plasma glucose >11.1 mmol/L during oral glucose tolerance test) [22].

### Follow-up and death ascertainment

Date of death for each patient was ascertained through record linkage with the Singapore National Registry of Births and Deaths. In Singapore, it is a statutory requirement that deaths are registered within 24 hours of their occurrence. Other investigators have previously validated completeness of outcomes ascertainment through record linkage in Singapore [23]. The last date of follow-up was March 1, 2012.

### Statistical analysis

Demographic and presenting characteristics between women and men were made using the chi-squared test for categorical data and the *t*-test for continuous age. Survival time is calculated from 30 days post admission for AMI to the date of all-cause death or date of censor. Survival curves were calculated by the Kaplan-Meier method. The associated hazard ratios (HR), corresponding 95% confidence interval (CI), and the effect of prognostic variables were estimated by Cox proportional hazards regression models. The changing pattern of the HR with time for women compared to men was assessed with a Cox model that included sex together with the interaction of sex and survival as a time-dependent covariate.

The interaction between sex and each of presenting clinical variables at admission, with and without adjustment for age, was assessed; the purpose being to determine the extent to which these factors individually modified the estimated HR for sex. This process identified age and diabetes as significant effect modifiers of the relationship between sex and mortality. As a consequence, the univariate sex model was extended to include age and diabetes and their interactions.

Given the significant interaction (diabetes by age), we further performed Cox models stratified by presence or absence of diabetes mellitus within age groups to quantify the risk of women. We applied a model that adjusts all pre-treatment variables and one that adjusts for both pre and in-hospital variables.

Age at admission was analysed in the following groups: 22-59, 60–69, 70-79 and 80-101 years. All P values were two-tailed with P < 0.05 regarded significant. STATA version 13 (College Station, Texas, USA) was used for all analyses.

## Results

After excluding non-residents and those under the age of 21 years, we identified a total of 15,151 patients who fulfilled criteria for AMI and were hospitalized between 1 January 2000 and 3 December 2005. Among them, 13,389 patients survived beyond 30 days after admission and were used in the analysis.

### Survival post 30 days

**Figure 1:**
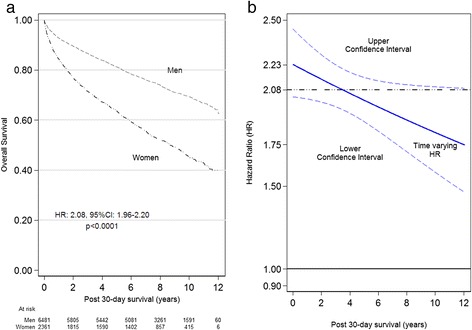
**Overall survival by sex and estimated change with time in HRs (a) Gender specific overall survival of patients following 30-days hospitalization for MI and (b) estimated change with follow-up time in the corresponding HR with associated upper and lower 95% confidence intervals (CI).**

Among the 13,389 patients, 3,420 (25.5%) women and 9,969 (74.5%) were men. Over the 12-year follow-up period with a median follow-up of 7.5 years, 1,761 deaths occurred among women (58.0% from cardiovascular death (CVD)) and 2,946 deaths occurred among men (56.2% from CVD). The corresponding survival curves showed a two-fold increased risk of death in women compared to men (HR: 2.08; 95% CI: 1.96-2.20, *P* < 0.0001) (Figure 1(a)). However, the magnitude of the relative hazard for women gradually tapered off with longer follow-up, with the highest excess mortality at discharge (HR: 2.23; CI: 2.04-2.45) and the lowest at 12 years (HR: 1.75; CI: 1.47-2.09) (Figure 1(b)).

### Possible risk factors

There are considerable differences between women and men in respect to some of the potential risk factors. Thus, as Table 1 shows, the median age of the women is 70 years compared to that of the men of 58. There are higher proportions of women with diabetes mellitus (51.4% vs. 31.4%), hypertension and renal failure. More women than men presented with non-ST-elevation myocardial infarction (NSTEMI) (62.3% vs. 51.1%) and with a higher Killip class (II–IV 37.3% vs. 25.2%). In all other respects, the genders are not too dissimilar. Women were less likely than men to receive reperfusion therapy and guideline-recommended drugs such as aspirin, *β*-blockers and lipid-lowering therapy.

**Table 1:** **Patient characteristics by sex of patients on hospital admission with acute myocardial infarction (AMI)**

**Presenting feature**		**Men**	**Women**	**All**	***P***
Number of 30-day survivors: n		9,969	3,420	13,389	
Ethnicity	Chinese	6,481 (65.0%)	2,361 (69.0%)	8,842	<0.001
	Malay	2,019 (20.3%)	655 (19.2%)	2,674	
	Indian	1,469 (14.7%)	404 (11.8%)	1,873	
Age (years)	Median	58	70	61	<0.001
	Range	22-101	27-100	22-101	
Age-group (years)	22-59	5,446 (54.6%)	675 (19.7%)	6,121	<0.001
	60-69	2,461 (24.7%)	954 (27.9%)	3,415	
	70-79	1,529 (15.3%)	1,151 (33.7%)	2,680	
	80-101	533 (5.4%)	640 (18.7%)	1,173	
Medical history	Prior MI	1,031 (10.3%)	359 (10.5%)	1,390	0.73
	Prior CABG	270 (2.7%)	98 (2.9%)	368	0.63
	Diabetes mellitus	3,128 (31.4%)	1,759 (51.4%)	4,887	<0.001
	Hypertension	5,212 (52.3%)	2,455 (71.8%)	7,667	<0.001
	Hyperlipidemia	4,178 (41.9%)	1,503 (43.9%)	5,681	0.025
	Renal failure	410 (4.1%)	283 (8.3%)	693	<0.001
MI category	STEMI	4,878 (48.9%)	1,288 (37.7%)	6,166	<0.001
	NSTEMI	5,091	2,132	7,223	
Killip class	I	7,460 (74.8%)	2,143 (62.7%)	9,603	<0.001
	II	1,752 (17.6%)	847 (24.8%)	2,599	
	III	568 (5.7%)	350 (10.2%)	918	
	IV	189 (1.9%)	80 (2.3%)	269	
Reperfusion therapy	Primary and Salvage PCI or Emergency CABG	4,524 (45.4%)	911 (26.6%)	5,435	<0.001
Medical therapy	Aspirin	9,096 (91.2%)	2,932 (85.7%)	12,028	<0.001
	*Β*-Blocker	7,806 (78.3%)	2,426 (70.9%)	10,232	<0.001
	Glycoprotein IIb/IIIa Inhibitors	775 (7.8%)	199 (5.8%)	974	<0.001
	Thienopyridine	5,829 (58.5%)	1,671 (48.9%)	7,500	<0.001
	ACE inhibitors	6,552 (65.7%)	2,249 (65.7%)	8,801	0.97
	Lipid-Lowering therapy	8,009 (80.3%)	2,518 (73.6%)	10,527	<0.001

MI = myocardial infarction; CABG = coronary-artery bypass grafting; STEMI = ST-elevation myocardial infarction; NSTEMI = non-ST-elevation myocardial infarction; PCI = percutaneous coronary intervention; ACE = angiotensin-converting enzyme.

### Interactions

Testing for interactions between sex and each presenting clinical feature at admission, we confirmed that age and diabetes were prominent effect modifiers on the relationship between sex and long-term mortality (Table 2). The increased risk of death in women compared to men decreased with increasing age; specifically, the HR decreased from 1.80 (CI: 1.52-2.13) for those aged 22-59, to 1.26 (CI: 1.11-1.42) for those 60-69, to 1.06 (CI: 0.96-1.17) and 0.96 (CI: 0.85-1.09), for those 70-79 and 80-101 years.

**Table 2:** **Interaction between sex and other admission characteristics of patients with acute myocardial infarction (AMI)**

				**Adjusted**	**Test for Interaction - with sex**	
**Presenting**		**Mortality (%)**		**HR (95% CI)**	**Unadjusted**	**Age-adjusted**
**feature**	**Categories**	**Women**	**Men**	**Women vs. Men**	***P***	***P***
Age (years)	22-59	24.3	14.2	1.80 (1.52–2.13)	<0.0001	–
	60-69	40.4	34.2	1.26 (1.11–1.42)		
	70-79	59.6	57.8	1.06 (0.96–1.17)		
	80-101	82.2	83.7	0.96 (0.85–1.09)		
Diabetes mellitus	Absent	42.0	24.2	0.99 (0.90–1.08)	0.036	0.040
	Present	59.9	40.8	1.12 (1.03–1.22)		
Prior MI	No	48.7	27.2	1.17 (1.09–1.25)	0.64	0.78
	Yes	74.4	47.5	1.19 (1.03–1.39)		
Prior CABG	No	51.0	28.9	1.16 (1.09–1.23)	0.021	0.99
	Yes	69.4	51.9	1.16 (0.87–1.55)		
Hypertension	No	46.1	23.5	1.18 (1.05–1.32)	0.0010	0.24
	Yes	53.5	34.8	1.09 (1.01–1.17)		
Hyperlipidemia	No	52.2	29.5	1.15 (1.04–1.26)	0.99	0.26
	Yes	48.3	26.5	1.24 (1.12–1.36)		
Renal failure	No	48.7	27.5	1.16 (1.08–1.24)	<0.0001	0.53
	Yes	80.2	70.0	1.09 (0.92–1.30)		
MI category	NSTEMI	55.7	33.6	1.17 (1.08–1.26)	0.96	0.28
	STEMI	44.6	25.3	1.09 (0.99–1.21)		
Killip class	I	43.1	23.5	1.18 (1.08–1.28)	0.0001	0.095
	II	60.7	41.9	1.12 (1.00–1.25)		
	III	75.7	64.3	0.94 (0.80–1.10)		
	IV	72.5	47.6	1.11 (0.80–1.54)		

MI = myocardial infarction; CABG = coronary-artery bypass grafting; NSTEMI = non-ST-elevation myocardial infarction; STEMI = ST-elevation myocardial infarction.

**Figure 2:**
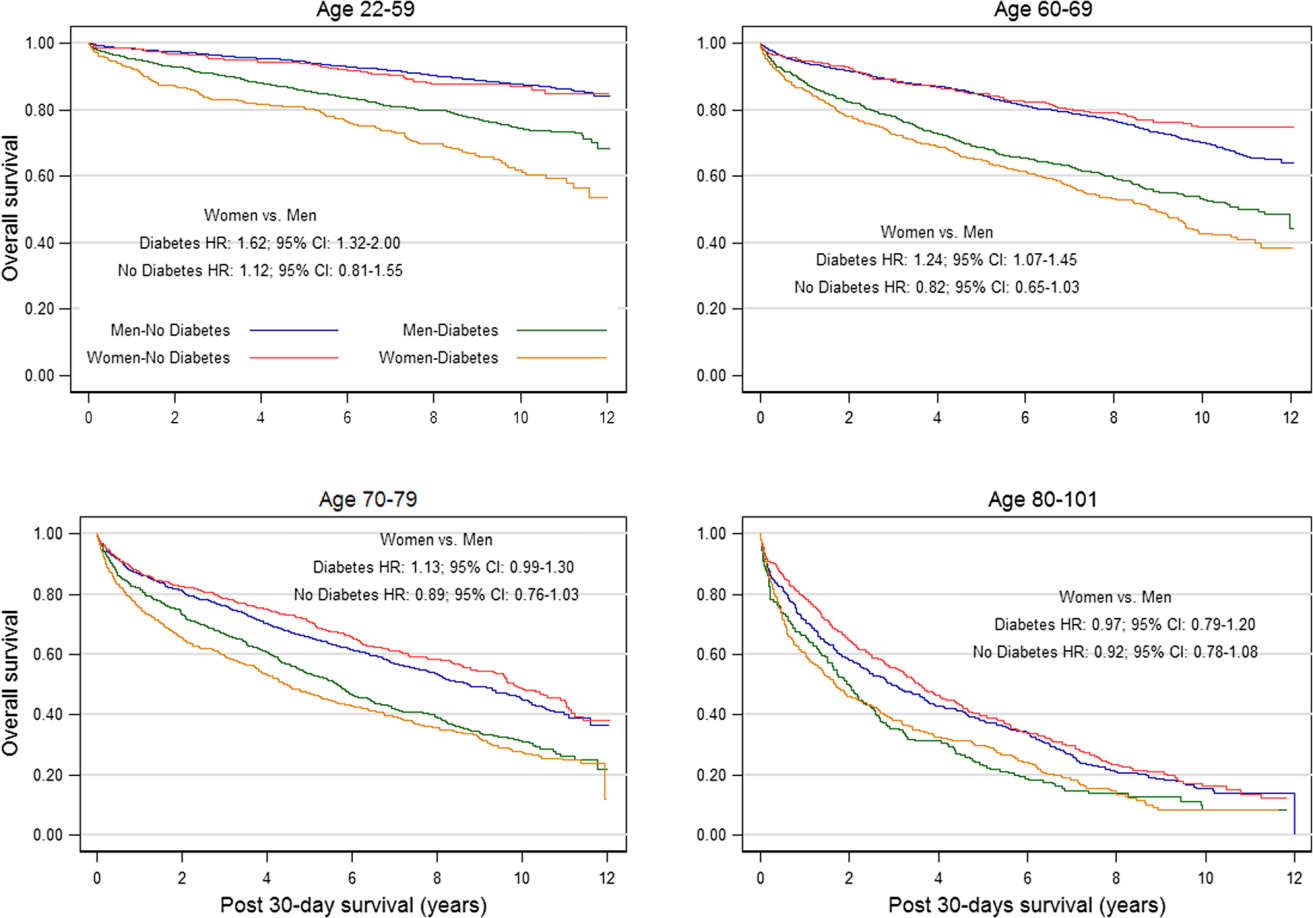
**Unadjusted Kaplan-Meier survival curves by sex and presence or absence of diabetes mellitus within age groups at hospitalization for acute myocardial infarction (AMI).**

After adjusting for age, there was a significant interaction between sex and diabetes (interaction *P* = 0.040). Women and men without diabetes had no significant difference in mortality (HR: 0.99; CI: 0.90-1.08). However, in the presence of diabetes, mortality was significantly higher in women than men (HR: 1.12; CI: 1.03-1.22). Survival curves for women and men for each age group by presence or absence of diabetes (Figure 2) indicate excess mortality in women as compared with men was most prominent in those aged 22-59 with diabetes (HR: 1.62; CI: 1.32-2.00; *P* < 0.0001).

### Multivariable analysis

To better characterize the joint association between age, sex and diabetes, we constructed regression model that includes the interactions of sex × diabetes, age × diabetes, and sex × age (Table 3). As compared to the model with sex and age alone with HR: 1.80 (CI: 1.52-2.13), the risk for younger women with diabetes is reduced to HR: 1.57 (CI: 1.31-1.87) after adding diabetics and the corresponding two-factor interactions which were all statistically significant (all *P* < 0.0001). Additional adjustment for ethnicity did not change these associations materially.

**Table 3:** Cox regression models with all two-factor interaction terms between sex, age and diabetes to examine their potential effect on the relative mortality hazard ratio (HR) of women compared with men*

	**HR (95% CI) Women vs. Men**			
	**Age (years)**			
**Diabetes**	**22-59**	**60-69**	**70-79**	**80-101**
Absent	1.22 (1.00–1.48)	0.92 (0.79–1.07)	0.88 (0.78–1.00)	0.85 (0.74–0.98)
Present	1.57 (1.31–1.87)	1.18 (1.04–1.35)	1.14 (1.01–1.28)	1.10 (0.94–1.28)

* P < 0.0001 for all interactions of sex × diabetes, age × diabetes, and sex × age.

**Figure 3:**
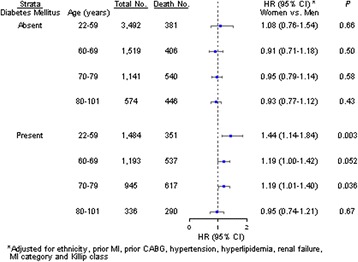
**Adjusted hazard ratio (HR) of women compared with men and its 95% confidence interval (CI) on long-term mortality by presence or absence of diabetes mellitus within age groups at hospitalization for acute myocardial infarction (AMI).**

In stratified analyses with multivariate adjustment (Figure 3), the prominently higher risk of death among women was significant among those diabetes younger than 60 years with adjustment of pre-treatment variables (adjusted HR: 1.44; 95% CI: 1.14 to 1.84; *P* = 0.003). The corresponding HR remains higher even after further adjustment for in-hospital treatment (adjusted HR: 1.32; 95% CI: 1.04 to 1.69; *P* = 0.024). The same comparison in older diabetic population (≥60 years) yielded an adjusted HR of only 1.12 (95% CI, 0.99 to 1.26). There were no differences between women and men in mortality rates in the subset of patients without diabetes.

## Discussion

The object of this study was to explore and quantify the possible adverse mortality experience of women against men following their survival post 30-days of hospital admission for AMI. This population-based study of unselected, consecutively registered Asian patients with AMI is notable for the following findings: First, a high proportion (51.4%) of Asian women with AMI had diabetes; this greatly exceeds the proportions reported in studies from Western populations (22.7% to 33.1% in Quebec, Canada [24]). Second, our study provided long-term (12 years) data showing that women were at twice the risk of death following an AMI compared with men, even after accounting for baseline differences, thereby extending results from prior studies with shorter lengths of follow-up [25]. Third, we found prominent joint associations between sex, age and diabetes on long-term survival following AMI, such that the excess long-term mortality risk in women was most pronounced in diabetic women under 60 years of age (>60% higher compared to diabetic men of similar age). These findings carry important implications for risk stratification among Asian patients with AMI, and suggest that young diabetic women should be identified as a high-risk group in need of better secondary prevention strategies.

Although previous studies have consistently shown that women with AMI have an increased risk of 30-day mortality [1-3,26-29], few studies have examined long-term outcomes. In an earlier analysis of 6,826 residents of Metropolitan Worcester, Massachusetts, USA, who survived hospitalization for AMI, Vaccarino et al. [25] found that sex-specific differences in 2-year mortality were confined to patients below 60 years of age. Unresolved issues included the extent to which female sex contributed to mortality risk, independent of other risk factors. Only a minority of previous studies addressed these issues, and data were limited to highly-selected clinical trial participants or single centers [30-35]. Most studies reported no difference in the long-term prognosis between men and women after accounting for age and other factors [34,35]. The exception is Meisinger et al. [36], which studied long-term mortality following AMI in 1,832 men and 611 women and reported, as we have now confirmed in Asian women, that it is higher only in diabetic females.

The remarkably large number of diabetic women in our cohort enabled an in-depth investigation. We showed that the long-term mortality risk associated with diabetes was most pronounced among younger women (<60 years). Further sex differences in long-term mortality diminished with increasing age and were no longer significantly different among older diabetic men and women. The prominent effect of age is consistent with the reversed sex effect in patients with diabetes from prior reports [4,24,37]. Data from North American patients with AMI [3] have further showed that sex differences in short-term mortality were confined to patients below 75 years of age, while older women tended to show relatively improved survival with prolonged follow-up, particularly when early deaths were excluded [38].

A common explanation for the greater mortality burden associated with diabetes in women is the higher prevalence of comorbidities in diabetic women who have experienced an AMI, as compared with men [36,39,40]. In Singapore, the majority of patients with type 2 diabetes are treated in the primary health care setting. Consistent with data from other regions, younger patients (<60) were found to have poorer glycemic control [41]. In our cohort, women with diabetes more frequently had a history of hypertension, renal failure and Killip class II–IV heart failure, compared with men with diabetes. However, careful adjustment for these comorbidities had a negligible effect on the sex estimates of mortality risk, both in the presence and absence of diabetes, suggesting that other factors may contribute to the greater mortality burden in women with diabetes. Postulated pathobiological derangements underlying sex differences in the association between diabetes and fatal CHD include sex hormone-related differences in systemic inflammation. It has been postulated that a heightened inflammatory state in young women may increase both the risk of diabetes and the risk of mortality following AMI [42,43].

Despite a high overall rate of use of aspirin, *β*-Blockers and lipid-lowering therapy among both men and women in our cohort, women with diabetes received relatively less evidence-based treatments. Studies have similarly shown that women with diabetes were less frequently treated with reperfusion therapy than men or women without diabetes [24,35] although disparities in treatment had no impact on the excess mortality in women [25,44]. Similarly, the observation of increased mortality among young diabetic women with AMI in our study remained robust even after correcting for sex-related treatment differences.

The strengths of our study include the availability of a large number of unselected patients with AMI who were consecutively entered into the population-based registry. Near complete ascertainment of deaths is possible in Singapore where all citizens and permanent residents are linked to the Singapore National Registry of Births and Deaths through a unique state-issued identification number. Since Asian populations appear to have an inherently higher burden of comorbidities, specifically diabetes and hypertension, as compared with Caucasians, the results of our study may be generalized to other Asian populations. Indeed, the prevalence of diabetes and hypertension among our Chinese, Malay and Indian ethnic groups in Singapore parallel the prevalence of diabetes and hypertension among these ethnic groups in the rest of Asia. To our knowledge, these data are the first to investigate the effect of sex and its links with age and diabetes on long-term mortality in a large national AMI registry of ethnically diverse women living in an industrialized Asian country.

The limitations of our study include the diagnosis of diabetes, which was either self-reported or ascertained through review of medical records. However, we anticipate the rate of undetected diabetes to be low as all inpatients of public hospitals are routinely screened for abnormalities of serum glucose as part of established clinical care pathways. We could not quantify post-discharge adherence to medication use and lifestyle changes such as smoking cessation following AMI. It is possible that the high death rate, mainly from CHD in younger women is due to less intensive risk factor management both before and after presentation with AMI, because of the general perception that young women are at a relatively lower risk of mortality.

## Conclusions

In conclusion, among Asian patients with AMI, one out of every two women had diabetes mellitus, with a twofold greater prevalence of diabetes in women than in men. These findings have important public health implications with respect to the prevention and treatment of diabetes in women. The greater post-MI mortality risk in women as compared with men persists at 12 years of follow-up. The excess risk of long-term mortality in women compared with men was most pronounced in diabetic women under 60 years of age. This high-risk group deserves better secondary prevention strategies to mitigate the long-term residual risk of mortality following AMI.

## Abbreviations

AMI: Acute myocardial infarction
CHD: Coronary heart disease
CVD: Cardiovascular death
SMIR: Singapore Myocardial Infarction Registry
CK-MB: Creatine kinase MB
HR: Hazard ratio
CI: Confidence interval
CABG: Coronary-artery bypass grafting
PCI: Percutaneous coronary intervention
ACE: Angiotensin-converting enzyme
STEMI: ST-elevation myocardial infarction
NSTEMI: non-ST-elevation myocardial infarction
